# Childhood HIV-associated nephropathy: 36 years later

**DOI:** 10.1007/s00467-020-04756-4

**Published:** 2020-10-12

**Authors:** Patricio E. Ray, Jinliang Li, Jharna R. Das, Pingtao Tang

**Affiliations:** 1 Department of Pediatrics, Child Health Research Center, University of Virginia School of Medicine, Room 2120, MR4 Building, 409 Lane Road, Charlottesville, VA 22908 USA; 2 Center for Genetic Medicine Research, Children’s National Hospital, Washington, DC 20010 USA; 3 The George Washington University Health Center, Washington, DC 20010 USA

**Keywords:** HIV nephropathy, Pediatric AIDS, Antiretroviral therapy, APOL-1, Heparan sulfate proteoglycans, HIV-*Tat*, Infection of podocytes, Kidney epithelial cells

## Abstract

HIV-associated nephropathy (HIVAN) predominantly affects people of African ancestry living with HIV who do not receive appropriate antiretroviral therapy (ART). Childhood HIVAN is characterized by heavy proteinuria and decreased kidney function. Kidney histology shows mesangial expansion, classic or collapsing glomerulosclerosis, and microcystic renal tubular dilatation leading to kidney enlargement. The pathogenesis of HIVAN involves the kidney recruitment of inflammatory cells and the infection of kidney epithelial cells. In addition, both viral and genetic factors play key roles in this disease. Modern ART has improved the outcome and decreased the prevalence of childhood HIVAN. However, physicians have had modest success providing chronic ART to children and adolescents, and we continue to see children with HIVAN all over the world. This article discusses the progress made during the last decade in our understanding of the pathogenesis and treatment of childhood HIVAN, placing particular emphasis on the mechanisms that mediate the infection of kidney epithelial cells, and the roles of cytokines, the HIV-*Tat* gene, and the Apolipoprotein-1 (*APOL1*) gene risk variants in this disease. In view of the large number of children living with HIV at risk of developing HIVAN, better prevention and treatment programs are needed to eradicate this disease.

## Introduction

**Fig. 1:**
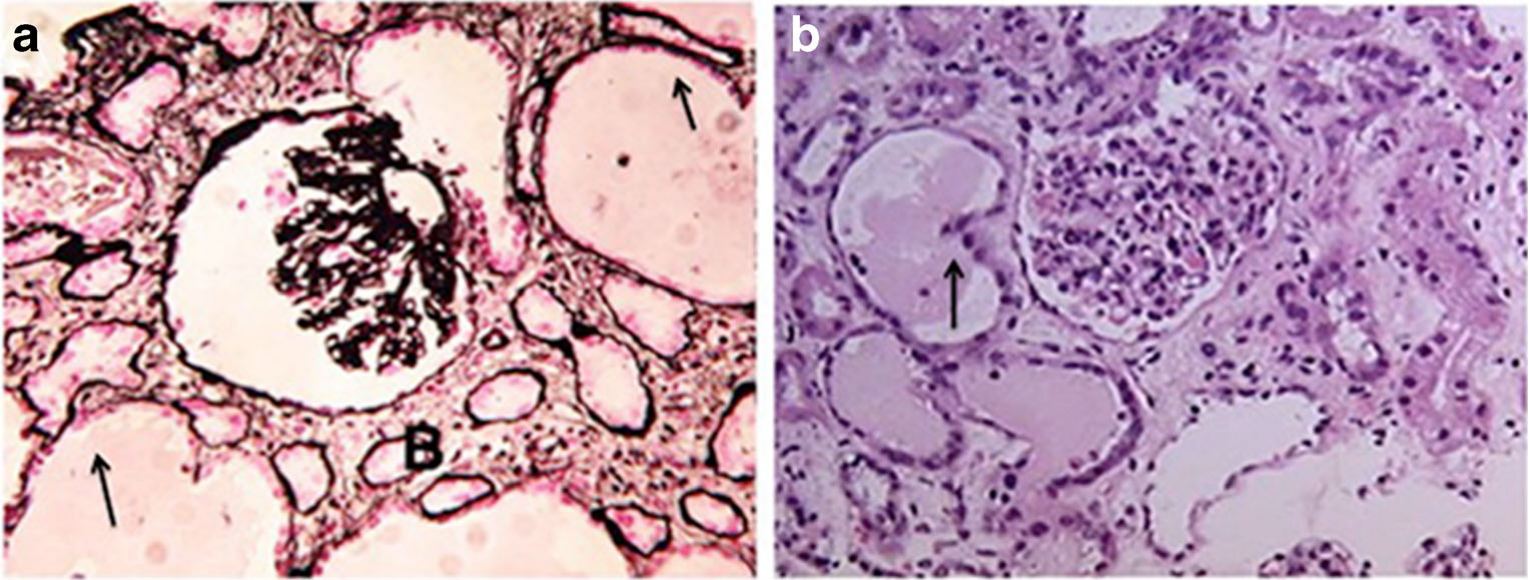
Panels (**a**) and (**b**) show representative kidney sections from children with HIVAN. Panel (**a**) shows a kidney biopsy with collapsing glomerulopathy stained with period acid Schiff. Panel (**b**) shows a kidney biopsy with mesangial expansion and enlarged glomeruli stained with H&E. Both panels show the tubular microcystic changes characteristic of childhood HIVAN (arrows). Original magnification × 200

HIV-associated nephropathy (HIVAN) was first reported in 1984 when a group of African-American adults infected with the human immunodeficiency virus-type 1 (HIV-1) presented with heavy proteinuria and rapid progression to kidney failure [1]. The identification of HIVAN in children provided key evidence to support the notion that HIV-1 per se was capable of inducing a new kidney disease, independently of other risk factors seen in adults [2]. The study of HIVAN attracted significant attention because it was a new kidney disease induced by a human retrovirus that was targeting people of African descent [1]. Childhood HIVAN is defined by the presence of proteinuria, often nephrotic syndrome, associated with mesangial hyperplasia and/or global-focal segmental glomerulosclerosis, and microcystic dilatation of renal tubules leading to kidney enlargement and failure [2–4] (Fig. 1). HIVAN bears a significant impact on the quality of life, treatment, and survival of HIV-infected children. Furthermore, it appears that once the clinical and renal histological features of HIVAN are well established, current treatments might not prevent its long-term progression to develop kidney failure. Therefore, it is necessary to improve our understanding of the pathogenesis and treatment of HIVAN in children. This article discusses the progress made during the last decade in our understanding of the pathogenesis and treatment of childhood HIVAN, focusing on the mechanisms that modulate the infection of kidney epithelial cells, and the role of cytokines, the HIV-*Tat* gene, and the Apolipoprotein-1 (*APOL1*) gene risk variants.

## What happens to children living with HIV today?

Despite the promising progress made in the HIV response during the last years, children under 15 years of age account for about 5% of all people living with HIV, 9% of new HIV infections and 13% of all AIDS-related deaths [5]. Those under 1 year of age are among those most vulnerable to HIV. If left untreated, they usually develop clinical symptoms in the first year of life, and by 1 year of age approximately one third of infected infants could die. Among the estimated 38 million people living with HIV in 2018, approximately 2.8 million were children under 19 years of age [6].

In countries where antiretroviral therapy (ART) has been successfully introduced, it has changed the face of HIV infection in a dramatic manner. Since 2010, new HIV infections among children have declined by 41%, but only half (54%) of all children living with HIV are getting treatment, and 100,000 children died of AIDS-related illnesses in 2018 (UNAIDS, Global AIDS update, 2019). The majority of these children acquired HIV-1 from their mothers during pregnancy, birth, or breast feeding. In addition, as of 2018, roughly 14.9 million children under the age of 18 had lost one or both parents to AIDS, and many have been affected by poverty, homelessness, and malnutrition. Modern ART, in combination with the avoidance of breastfeeding and good access to HIV and pregnancy care services, has reduced the risk of mother-to-child HIV transmission to 1-2% [7]. HIV-infected infants now survive to adolescence and adulthood, but they need to receive daily effective ART treatment to remain in good health. However, as discussed above, these therapies are still not widely accessible in most resource-limited countries, where the burden of HIV-infection is very high.

African Americans represent approximately 65% of all children with HIV-1 infection or AIDS in the USA. In addition, over 80% of the estimated number of children under 15 years of age living with HIV reside in sub-Saharan Africa [5]. Since HIVAN is seen almost exclusively in Black children, the majority of children living with HIV are at higher risk of developing HIVAN if they are not treated appropriately with ART. However, providing chronic ART and good medical care to children living with HIV remains a big challenge all over the world. Unfortunately, the true prevalence of childhood HIVAN is unknown since in many pediatric centers, kidney biopsies have not been performed regularly. Studies done in the early years of the AIDS epidemic reported a prevalence of childhood HIVAN of approximately 10–15% in populations with a majority of Black children [2, 3]. Subsequently with the development of highly effective ART, the prevalence of HIVAN decreased significantly, but we continue to see children who develop HIVAN because they do not receive appropriate ART. In addition, persistent proteinuria, which could be the first clinical sign of HIVAN, is common in children living with HIV on ART, and the prevalence of proteinuria in these patients ranges from 5 to 10% [4, 8]. Table 1 describes epidemiological, clinical, diagnostic, histological, and laboratory features typical of children and adolescents with HIVAN.

**Table 1:** Typical features of HIVAN in children from the Washington, D.C. area

*Epidemiology* Almost exclusively seen in Black children and adolescents Majority acquired HIV-1 through vertical transmission (~ 97%) Clinical onset: any time after 1 year of age, more frequent during late childhood and adolescence Both sexes In children from other ethnic groups, suspect other kidney diseases In children with consistently suppressed viral load, suspect other kidney diseases	
*Early clinical and laboratory diagnosis* Requires a high index of suspicious Mild or moderate persistent proteinuria A dipstick proteinuria 1+, in the absence of acute infection episodes, requires a quantitative assessment of proteinuria and clinical follow-up Normal BUN and SCr levels Normal blood pressure Abnormal urinary sediment, casts, shedding of renal epithelial cells If significant hematuria, elevated SCr, or hypertension are present initially, suspect other kidney diseases	
*Late clinical and laboratory diagnosis* Gross proteinuria, nephrotic syndrome Abnormal urinary sediment, cast, shedding of renal epithelial cells and microcysts High BUN and SCr levels Hypertension Hyperlipidemia Fluids—electrolyte and salt wasting disorders Sonogram: enlarged echogenic kidneys	
*Renal histology* A kidney biopsy is needed to confirm the diagnosis of HIVAN. “It is not always HIVAN” Glomerular enlargement, mesangial hyperplasia Classic or collapsing focal glomerulosclerosis Microcystic tubular dilatation Tubulointersitial injury and recruitment of inflammatory cells	
*Course* Usually associated with other AIDS symptoms Depends on adherence and response to ART Mild, moderate, or gross persistent proteinuria Slow progression to kidney failure depends on the stage of diagnosis and ART response Early diagnosis and treatment may reverse the progression of HIVAN A late diagnosis or treatment is unlikely to reverse the progression Death from other AIDS-related complications	

*HIVAN* HIV-associated nephropathy, *BUN* blood urea nitrogen, *SCr* serum creatinine, *ART* antiretroviral therapies

### Pathogenesis of childhood HIVAN

#### Lessons from HIV-transgenic (Tg) mice

Our understanding of the pathogenesis of HIVAN has evolved throughout the years in correlation with the rapid progression made in the field of HIV. A major early breakthrough was made at the National Institutes of Health in 1991, with the generation of HIV-1 transgenic (HIV-Tg_26_) mice that developed a HIVAN-like disease [9]. This mouse model was developed with a replication defective HIV construct driven by the HIV-LTR but lacking a 3-kb sequence overlapping the *gag* and *pol* HIV genes that are essential for viral replication [9]. Despite the limited activity of the HIV-*tat* gene in rodents, which is necessary for HIV transcription, these mice express HIV-1 mRNA in a wide range of tissues, including kidney glomerular and tubular epithelial cells [10]. HIV-Tg rats generated with the same HIV-*Δgag/pol* transgene [11] show a similar kidney localization of HIV-genes, and develop the typical histological features of childhood HIVAN, including mesangial hyperplasia, visceral epithelial cell enlargement, glomerular sclerosis, tubular cell proliferation, and tubular microcystic changes. One distinguishing feature of the HIV-Tg rat model, however, is that the HIV-transgene is more widely expressed, and the HIV protein gp120 is detected in the circulation and mononuclear cells [11]. These findings suggest that HIV-*tat* may be more active in rats. Overall, both HIV-Tg mouse and rat models demonstrate that the expression of HIV genes in kidney epithelial cells play a critical role in the pathogenesis of HIVAN. In addition, many other HIV-Tg mouse models, which were described in detail in a previous review [12], highlighted the role of two accessory HIV genes, *nef* (Negative regulatory factor) and *vpr* (viral protein R), as key determinants in the pathogenesis of HIVAN in these mice. It is worth mentioning, however, that not all HIV-Tg mice or rats that express HIV genes in the kidney develop kidney disease. Furthermore, when F1 hybrids of HIV-Tg_26_FVB/N were crossbred with five other inbred mouse strains, there were striking variations in the kidney phenotype of HIV-Tg_26_ mice [13]. These studies demonstrate that a strong genetic influence modulates the outcome of HIVAN in mice, and suggest that other still unknown genetic and/or environmental factors may be needed to develop the complete HIVAN phenotype in mice.

#### Infection of kidney epithelial cells

**Fig. 2:**
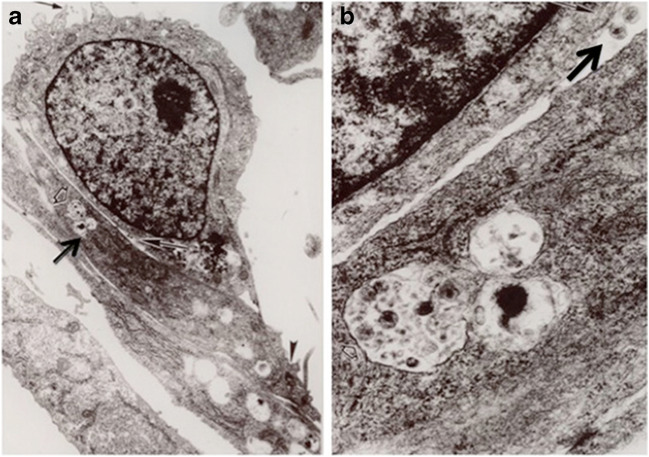
Panels (**a**) and (**b**) show a mononuclear cell on top of renal tubular epithelial cells (RTEC) cultured from a child with HIVAN. The arrowhead in panel (**a**) points to a tight junction between RTEc. The arrow points to HIV viral particles inside RTEc. Panel (**b**) shows a higher magnification of the viral particles inside RTEc, as well as viral particles in the space between mononuclear and RTEc (arrow). Original magnification ×10,000 (**a**) and ×50,000 (**b**)

Since kidney epithelial cells do not express the CD4 molecule, which is the major HIV-1 receptor, a major controversy in the pathogenesis of HIVAN is whether these cells can become productively infected. HIV-1 induces a productive infection of T cells mainly by a process that involves the fusion of its envelope protein (gp120-gp41) to the plasma membrane [14], which is triggered by the interaction of gp120 with the CD4 molecule in collaboration with the HIV-1 co-receptors CXCR4 or CCR5. In 1998, kidney epithelial cells cultured from the urine of children with HIVAN were used for the first time to demonstrate that HIV-1 isolates derived from children with HIVAN can induce a low-level productive infection in the absence of CD4 [15]. These tubular epithelial cells produced low levels of p24 antigen when compared with HIV-infected macrophages, but were able to transfer viruses and infected co-cultured HIV-negative mononuclear cells even after 20 days in culture [15]. In addition, the present study showed that cell-to-cell contacts between HIV+ mononuclear cells and kidney epithelial cells is the most efficient mechanism to infect the latter cells [15] (Fig. 2). Subsequent studies confirmed the presence of viral transcripts in podocytes and kidney tubular epithelial cells in kidney sections derived from patients with HIVAN [16]. Nonetheless, other studies done in podocytes and kidney tubular epithelial cells cultured from HIV-negative people concluded that these cells were not productively infected [17, 18]. In contrast, another study confirmed that interactions between infected T cells and kidney tubular epithelial cells create virological synapses that allow viral uptake and gene expression in kidney epithelial cells [19].

In any case, many studies support the notion that HIV-1 can enter CD4 negative kidney epithelial cells via endocytosis. Our previous infection studies done in primary kidney tubular epithelial cells cultured from children with HIVAN showed HIV-1 particles in intracellular compartments [15] (Fig. 2). In addition, although the endocytic uptake of HIV particles usually leads to a non-productive infection when viruses are degraded in the lysosomes, it is possible that some viruses that are internalized via endocytosis prior to membrane fusion can complete its fusion process within endosomes or escape into the cytosol [20]. Dynamin, a 100-kDa GTP-ase that mediates the release of endocytic vesicles from the plasma membrane, can facilitate the escape of HIV-1 from endosomes [20]. In contrast, dynasore, a small molecule inhibitor of the dynamin GPT-ase activity, prevents the scission of endocytic vesicles from the inner leaflet of the plasma membrane and inhibits clathrin-mediated endocytosis, as well as the fusion of HIV-1 to endosomes [20]. Taken together, these findings imply that cells could potentially become productively infected, although at low levels, via endocytosis through an envelope glycoprotein-dynamin-dependent fusion within intracellular compartments [20]. HIV-1 infection via endosomes has been reported in macrophages, lymphocytic cells, and HeLa cells [21], and two studies showed that HIV-1 enters cultured human podocytes via dynasore-mediated endocytosis [18, 22]. In summary, HIV is efficiently taken up by clathrin-coated vesicles [23] and dynamins can facilitate the release of these vesicles from the plasma membrane and endosomes.

#### Role of trans-membrane TNF-α enhancing the infection of cultured kidney epithelial cells

**Fig. 3:**
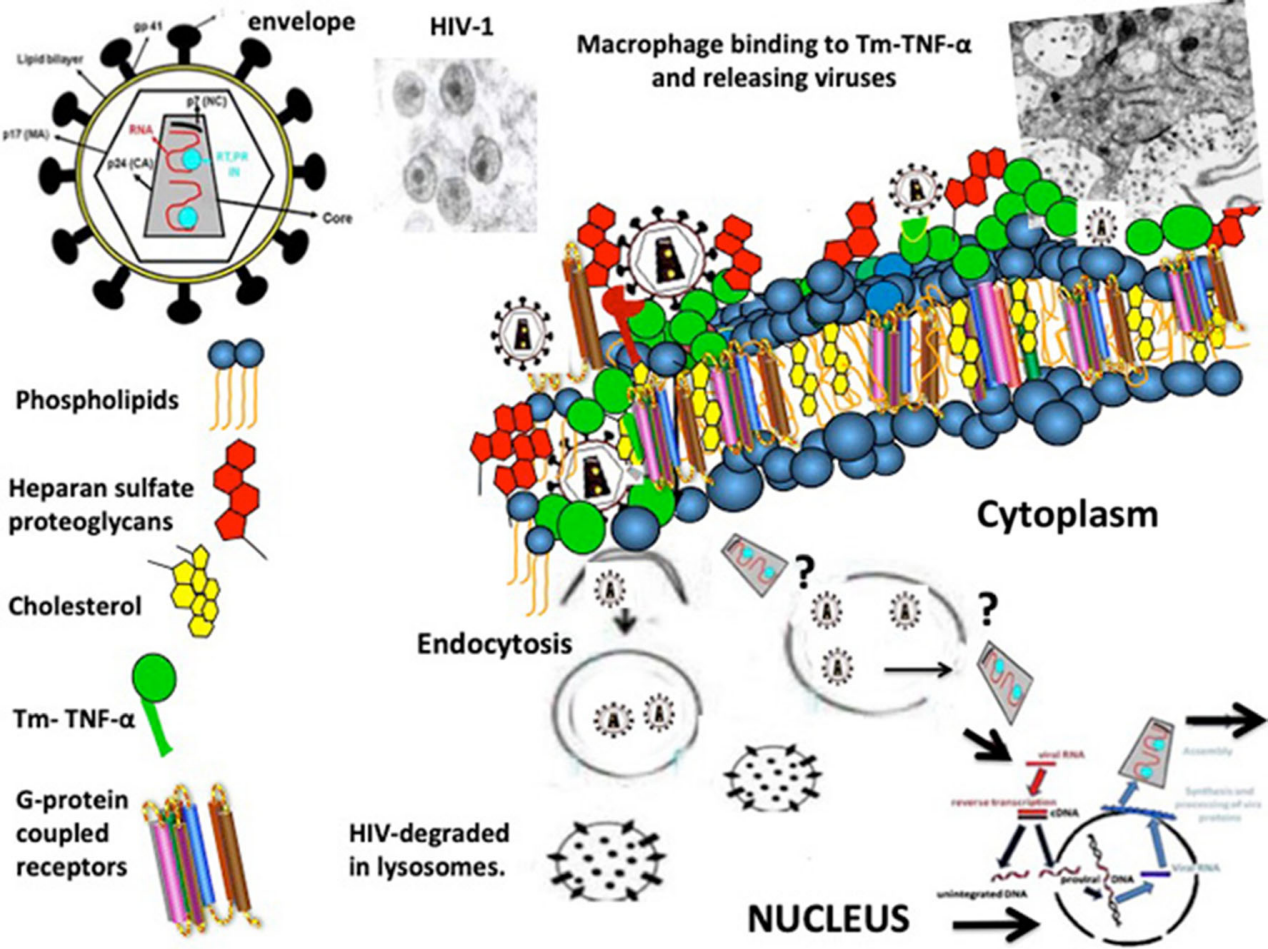
Proposed mechanisms for explaining the infection of kidney epithelial cells cultured from children with HIVAN. Transmembrane-TNF-α (Tm-TNF-α) expressed on the surface of kidney epithelial and mononuclear cells facilitates the binding of HIV-infected mononuclear cells to kidney epithelial cells. Viruses are released into a “virological synapse” created between these cells (Fig. 2). HIV is internalized via an envelope-clathrin-mediated endocytosis mechanism prior to membrane fusion. Heparan sulfate proteoglycans facilitate the binding and entry of HIV-1 via endocytosis. Once inside endosome-like compartments, viral particles can be degraded in endolysosomal compartments (non-productive infection). A small number of virus particles may undergo fusion in endosome-like compartments or escape into the cytosol through other mechanisms that are not clearly understood at the present time (?). Viral particles released into the cytoplasm undergo reverse transcription and integration into the nucleus. In turn, after undergoing several assembling steps, new infectious viral particles can be released

In 2017, we described a new role for transmembrane TNF-α (tm-TNF-α), facilitating the infection of CD4-negative podocytes and tubular epithelial cells cultured from children with HIV-associated kidney diseases [22]. More specifically, we found that tm-TNF-α expressed in podocyes cultured from children with HIVAN, increases the viral entry process via a dynamin-mediated mechanism that is blocked by dynasore, and enhances the replication of HIV-1 in these cells via an NF-kB-mediated mechanism [22]. Heparan sulfate proteoglycans (HSPG) also play a critical role in this process, enhancing the attachment and entry of the HIV-1 envelope protein [22]. After viral entry, the precise mechanisms through which tm-TNF-α facilitates the release of HIV-1 into the cytoplasm and its integration in the nuclei of podocytes remain undefined. In this regard, it is worth mentioning that tm-TNF-α is the precursor of soluble TNF-α, and can act both as a ligand or as a receptor [24]. Therefore, tm-TNF-α can induce cell-to-cell contact-dependent signaling and also raise the concentration of soluble TNF-α in the proximity of TNF receptors (TNFR) [24]. These events enhance the expression of cell adhesion and signaling molecules involved in viral entry and replication. Furthermore, tm-TNF-α activates the non-canonical NF-kB pathway acting through TNFR2, which has higher affinity for tm-TNF-α than soluble TNF-α [24, 25]. Finally, through all these mechanisms, tm-TNF-α may facilitate the formation of viral synapses between mononuclear and kidney epithelial cells [15, 19] (Fig. 3). Taken together, these findings suggest that kidney epithelial cells may be “primed” by tm-TNF-α to become latently or productively infected, albeit at low levels, when they interact with HIV-infected mononuclear cells. Furthermore, studies in homozygous HIV-Tg_26_ mice showed that high circulating levels of TNF-α precipitate the death of these mice, and this process can be ameliorated with anti-TNF-α antibodies [26]. In addition, TNF-α appears to worsen the progression of HIVAN in heterozygous Tg_26_ mice, probably by enhancing the expression of HIV-genes and inducing podocyte injury [27]. In summary, since high levels of TNF-α are detected in the plasma of HIV+ children [28], these studies suggest that TNF-α may play an important role in the pathogenesis of childhood HIVAN.

#### Novel roles of HIV-tat in childhood HIVAN

The HIV-Tat protein is a powerful transcriptional factor encoded by two exons. The first exon encodes the activation domain, which interacts with cyclin T1, and the basic domain (amino acids 49–57), which is required for the nuclear localization of Tat [29]. The second exon encodes the RGD motif (C-terminal amino acids 73–86), which enhances the angiogenic activity of Tat acting through integrin receptors [30]. Tat plays an essential role in HIV replication by recruiting a cellular human protein called cyclin T1, which efficiently increases viral transcription, and also induces the activation of NFκB and the HIV-LTR [31]. Cloning and characterization of the murine CycT1 protein revealed that mouse cyclin 1 lacks a critical cysteine residue that is needed to form a complex with HIV-1 Tat and increase HIV-1 transcription [32]. Because both the infection and HIV-replication processes are bypassed in HIV-Tg mice, and murine cyclin 1 has weak interactions with Tat, transgenic mice are not the ideal experimental model to elucidate the role of HIV-Tat in the pathogenesis of HIVAN.

**Fig. 4:**
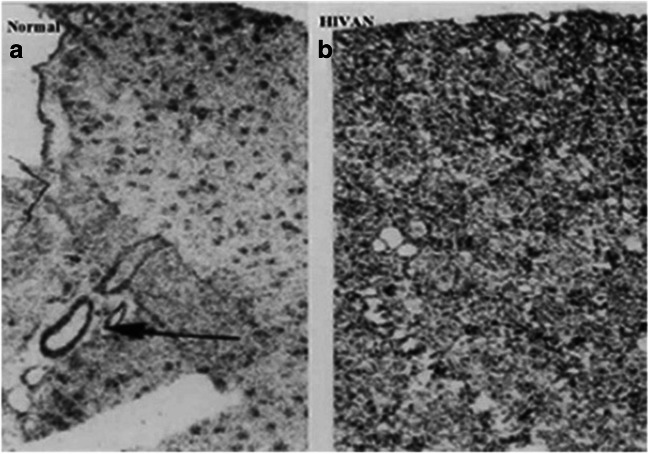
Total binding of ^125^I-Fibroblast Growth Factor-2 to heparan sulfated proteoglycans (HSPG) located in glomeruli, vessels, and the kidney interstitium of children with and without HIVAN. Panel (**a**) shows a representative kidney section from a control child (normal). The arrow in panel (**a**) points to ^125^I-FGF binding to kidney vessels. Panel (**b**) shows increased binding of ^125^I-FGF to HSPG in the kidney cortical interstitium in a child with HIVAN. Original magnification ×60. (Figure is reproduced with permission of Springer from Ray PE, Liu Xu, Rakusan T, Liu Xue-Hui. “A 20-year history of childhood HIV-associated nephropathy” *Pediatric Nephrology* 19: 1075–1092, page 1083; License Number 3984431229899)

**Fig. 5:**
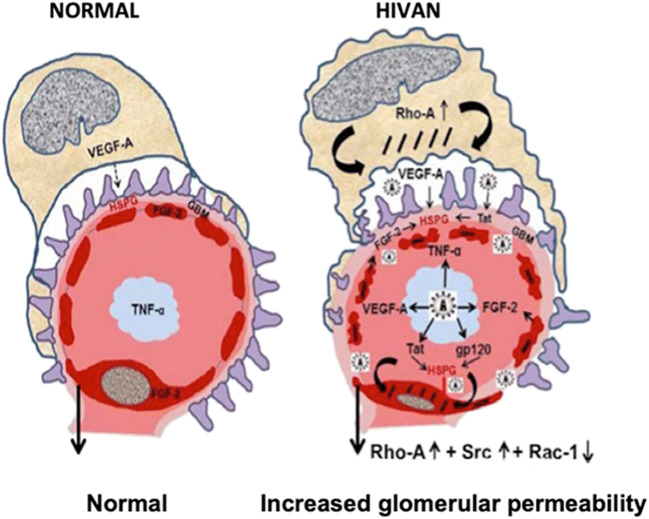
HIV-Tat, fibroblast growth factor-2 (FGF-2), and vascular endothelial growth factor (VEGF-A) increase the permeability of the glomerular filtration barrier. Under normal conditions, VEGF-A released from podocytes is needed to maintain the normal structure and survival of endothelial cells. FGF-2 is not released from healthy endothelial cells, and very low levels of TNF-α are released from mononuclear cells. In contrast, during the early stages of HIVAN, HIV-Tat and other heparin binding cytokines or viral proteins released by HIV-infected mononuclear cells, injured endothelial cells, or podocytes affect the phosphorylation and activity of Rho-A, Src, and Rac-1 in podocytes and glomerular endothelial cells. These changes alter the cytoskeleton structure of podocytes and glomerular endothelial cells and increase the permeability of the glomerular filtration barrier causing proteinuria

One characteristic feature of HIVAN in children is the up-regulation of renal heparan sulfate proteoglycans (HSPG) (Fig. 4). HIV-Tat and other heparin binding growth factors released by infected cells can interact with negatively charged HSPG expressed in glomerular, tubular epithelial, and kidney interstitial cells [33]. In this manner, extracellular Tat can act as a cytokine [34], entering cells via endocytosis mediated by HSPG, and increasing the replication of HIV-1 via TNF-α and NF-κB activation [35]. In addition, in recent years we found that Tat is preferentially recruited to lipid rafts (LR) in podocytes cultured from children with HIVAN [36]. LRs are specialized membrane domains enriched in certain lipids, cholesterol, and proteins that serve as docking sites for signaling proteins, including G protein–coupled receptors [37]. Actin binding proteins bind to polyphosphoinositides located in LRs, and these proteins link the actin cytoskeleton with signaling molecules that are enriched in the LRs [38]. In fact, activation of G protein–coupled receptors by HIV-Tat in endothelial cells activates Rho-kinase and the myosin binding subunit of the myosin light chain (MLC) [39]. Rho-A regulates the dynamic organization of contractile actin–myosin filaments and stress fiber formation through activation of Rho-kinase and MLC [40]. These molecules play an important role maintaining the cytoskeletal structure of podocytes as well as the permeability of the glomerular basement membrane (Fig. 5). Analogous changes in Rho-A activation were induced in the kidney of HIV-Tg_26_ mice infected with adenoviral Tat vectors, and they precipitated the development of HIVAN [36]. In a similar manner, previous studies showed that activation of the Rho-A pathway specifically in podocytes causes chronic kidney failure in transgenic mice [41]. Furthermore, the Tat basic domain, which contains a cluster of basic residues (RKKRRQRRR) that are known to carry proteins and DNA molecules across the cell membranes, appears to be essential for the recruitment of Tat to LRs [36], and to modulate the activity of extracellular Tat in human podocytes [29, 36]. Three arginine residues in the basic domain are essential for the stable association of Tat with LRs and Tat’s signaling interactions with heparin binding growth factors in podocytes cultured from children with HIVAN [36].

Finally, matrix metalloproteinases (MMP) can also modulate the activity of Tat. Children with HIVAN excrete high levels of MMP in the urine, in correlation with the progression of their kidney disease [42]. MMP-9 belongs to a family of zinc binding endopeptidases that degrade extracellular matrix proteins, including HSPG and collagen [43], and facilitates the release of Tat and Fibroblast Growth Factor-2 (FGF-2) bound to HSPG [44]. We found that MMP-9 was localized in LRs in human podocytes, and that the basic domain of Tat increased the expression of MMP-9 in LRs [36]. Furthermore, the substitution of six arginines in the Tat basic binding domain for alanines prevented the association of Tat with LRs, impaired its ability to enhance FGF-2 signaling or MMP-9 expression in cultured podocytes, and failed to induce severe kidney disease in HIV-Tg_26_ mice [36]. Taken together, these data suggest that the Tat basic binding domain could become a new target to develop therapies against childhood HIVAN.

#### Renal proliferative lesions and childhood HIVAN

**Fig. 6:**
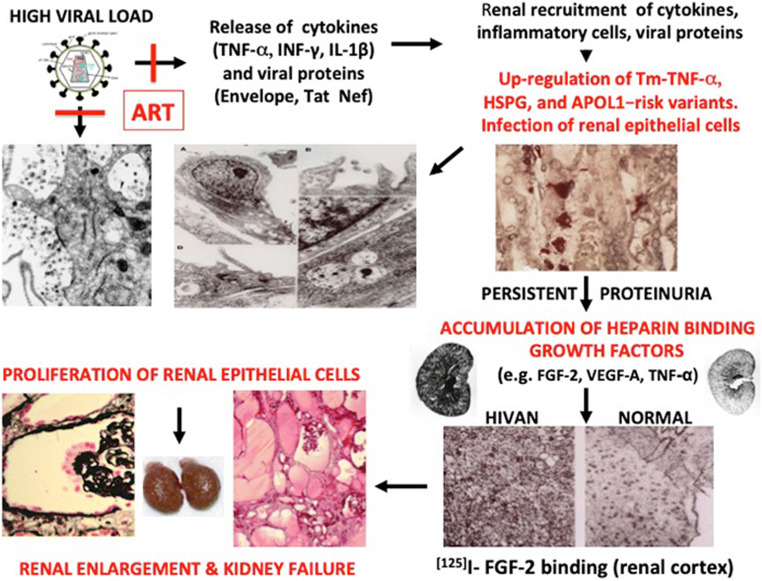
Proposed mechanisms for explaining the pathogenesis of childhood HIVAN. A high viral load increases the release of cytokines and viral proteins in the circulation. Cytokines, viral proteins, and inflammatory cells are recruited in the kidney, increasing the expression of Tm-TNF-α, heparan sulfate proteoglycans, and APOL1 in kidney epithelial, endothelial, and tubulointerstitial cells. All these changes induce glomerular and tubulointerstitial injury, increasing the recruitment of inflammatory cells, and facilitating the infection and injury of more kidney epithelial cells causing persistent proteinuria. The accumulation of heparin binding growth factors and viral proteins increase the proliferation of podocytes and tubular epithelial cells leading to kidney enlargement and kidney failure. Antiretroviral therapy (ATR) blocks the early stages and development of HIVAN, but may not affect the secondary proliferative changes once the kidney damage is established

As discussed before, HIV-Tg_26_ mice provided an important clue to understand the pathogenesis of the kidney epithelial proliferative changes characteristics of HIVAN. Briefly, the natural history of the kidney disease in HIV-Tg_26_ mice can be divided in two different stages [33]. The first stage is associated with an up-regulated expression of HIV genes in kidney epithelial cells and the onset of proteinuria. No direct kidney epithelial proliferative changes are noted at this stage [33]. In patients with a high viral load, the first stage involves the kidney recruitment of cytokines, viral proteins, and inflammatory cells that injure podocytes and tubular epithelial cells (Fig. 6). The second stage of HIVAN in HIV-Tg_26_ mice, is characterized by glomerular and tubular epithelial proliferative changes, and is associated with lower levels of HIV gene expression and the accumulation of heparin binding growth factors bound to renal HSPG (Fig. 6). It is unclear whether the proliferating cells seen in Bowman’s space are de-differentiated podocytes, parietal epithelial cells, or progenitor epithelial cells. The tubular microcystic changes are composed of flattened epithelial cells that express very low levels of HIV genes [33]. These more primitive kidney epithelial cell types appear to have a limited transcriptional activity to support HIV gene expression. Podocytes or tubular epithelial cells cultured from the urine of children with HIVAN do not proliferate when exposed to HIV [15]. Taken together, these data do not support the notion that HIV-1 genes induce direct proliferative changes in kidney epithelial cells cultured from children. In contrast, previous studies done in cultured murine epithelial cells immortalized with the SV-40 large T antigen showed that HIV-Nef increases the proliferation of these cells [45]. However, these findings were not reproduced in primary human kidney epithelial cells.

As discussed above, the kidney accumulation of heparin binding growth factors appears to play a critical role inducing the de-differentiation and proliferation of podocytes and tubular epithelial cells [33, 46, 47]. Podocytes are terminally differentiated cells, and when they are forced to proliferate, they can detach, die, or undergo apoptosis [48, 49]. These cells, however, can be replaced by parietal epithelial cells or renal progenitor cells [50, 51], which are sensitive to FGF-2 and other cytokines [52]. In addition, we found that FGF-2 and VEGF-A potentiate the ability of HIV-Tat to induce cytoskeletal changes and increase the permeability of cultured podocytes and endothelial cells [53]. Finally, the urinary levels of FGF-2 and VEGF-A are elevated in children with HIVAN, and these cytokines appear to be good candidate biomarkers to follow the outcome of childhood HIVAN [42, 53]. Current studies are underway to determine the sensitivity and specificity of these heparin binding growth factors to follow the outcome of children with HIVAN. Children may be more sensitive to the kidney accumulation of FGF-2 because their kidneys are growing, and they have high expression levels of HSPG, MMPs, FGF receptors, and higher plasma and tissue levels of FGF-2 compared with adults [54].

#### Apolipoprotein-1 and host genetic susceptibility to develop HIVAN

People of African descent are at markedly increased risk for developing HIVAN. The discovery in 2010 that two common genetic variants of the *APOL1* gene, named G1 and G2, located in a region of chromosome 22q12 were strongly associated with the risk of developing HIVAN, constituted a major breakthrough in understanding why mainly people of African descent develop HIVAN [55, 56]. In sum, two haplotypes harboring three coding sequence mutations of *APOL1* were identified as risk variants. The first one, termed G1, is a two-non-synonymous-SNP haplotype (rs73885319 (A → G; S342G) and rs60910145 (G → T; I384M)). The second one, termed G2, is a two-codon-deletion haplotype (rs71785313 (6 bp in frame deletion; DN388Y389)). The APOL1 risk variants increase the lifetime risk for people with untreated HIV+ to develop HIVAN by ~ 50% [57]. It should be noted, however, that a significant number of HIV+ children of African descent develop HIVAN independently of the APOL1 risk variants [58, 59]. In fact, previous studies done in the USA suggest that the APOL1 risk variants may play a more relevant role in adults when compared with young children [59, 60]. Thus, it is possible that other unknown genetic factors may play an additional role precipitating HIVAN in Black children. However, a recent study in African children living with HIV found a 22-fold increase odds of albuminuria (≥ 30 mg/g) in those carrying the high-risk APOL1 genotypes when compared with children carrying the low-risk genotype [61].

The effects of the APOL1 risk alleles in kidney epithelial cells have been discussed in depth in previous studies [62–64]. Briefly, APOL1 has been localized in several tissues, including podocytes, tubular epithelial cells, endothelial, and vascular smooth muscle cells [65]. Unfortunately, despite extensive research efforts, we still do not have a good understanding of how the APOL1 risk variants predispose to the development of HIVAN. However, several studies done in cultured kidney cells [62–64], transgenic mice [66, 67], and transgenic flies [68, 69] have provided relevant information. In general, all these studies show that the APOL1 G1 or G2 risk variants, when overexpressed, are more toxic than the wild-type APOL1. In addition, the endogenous expression of APOL1 is induced by TNF-α and IFN-γ [62, 70], two cytokines released by HIV-infected cells. These findings are consistent with the hypothesis that HIV acts as a second hit, enhancing the toxicity of the APOL1 risk variants in podocytes, rather that inducing direct proliferative changes in these cells. In turn, injured podocytes may be replaced by proliferating parietal epithelial cells or progenitor cells, and this process is more likely to be driven by cytokines and heparin binding growth factors accumulated in the kidney (Fig. 6).

#### Treatment of HIVAN

ART has substantially changed the face of children living with HIV-associated kidney diseases. In a large multi-center trial evaluating long-term outcomes in children living with HIV, the death rate attributable to CKD was ~ 2% [71]. However, children living with HIV need to be enrolled in effective chronic treatment programs to stay healthy, and providing continuous acute and chronic care to these patients is a major challenge today. Obstacles to treating all pediatric patients include the lack of screening or simple diagnostic tests, insufficient understanding of the beneficial effects of ART by the parents, and the cost of pediatric ART formulations. In resource-limited countries, even children on ART show poor clinical outcomes [72]. In these cases, additional efforts are needed to make antiretroviral drugs available to more children, and to develop simple treatment strategies to assure the long-term adherence to these treatments. Most countries have moved or are moving to provide lifelong ART regardless of CD4 cell count to all pregnant and breastfeeding women, and many are moving to implement viral load testing as the preferred means of monitoring people who are taking ART. New point-of-care viral load testing technologies offer further potential to expand this approach. Further, safer and more efficacious antiretroviral drugs are becoming available and a newer class of drugs—integrase inhibitors—is becoming more affordable for low- and middle-income countries.

More than 25 antiretroviral (ARV) drugs in six mechanistic classes are Food and Drug Administration (FDA) approved for treatment of HIV infection. These six classes include the nucleoside/nucleotide reverse transcriptase inhibitors (NRTIs), non-nucleoside reverse transcriptase inhibitors (NNRTIs), protease inhibitors (PIs), a fusion inhibitor (FI), a CCR5 antagonist, and integrase strand transfer inhibitors (INSTIs). In addition, two drugs, ritonavir (RTV) and cobicistat (COBI), are used solely as pharmacokinetic (PK) enhancers (i.e., boosters) to improve the PK profiles of some ARV drugs [73]. The initial ARV regimen for a treatment-naive adult or adolescent patient generally consists of two NRTIs, plus a drug from one of three drug classes: an INSTI, an NNRTI, or a PK-enhanced PI. A discussion related to the selection of the appropriate antiretroviral (ARV) drug treatments and the potential effect of these drugs on the kidney is beyond the scope of the present review. Table 2 describes important issues to take into consideration for the treatment of children and adolescents with HIVAN. Briefly, the kidney excretes all NRTIs except for abacavir, and their dosage should be adjusted according to the estimated glomerular filtration rate (eGFR). Other classes of ARV drugs (NNRTI, PI, fusion inhibitor, integrase, chemokine receptor antagonists) do not undergo significant kidney excretion and do not require adjustments in patients with kidney insufficiency. In all cases, nephrologists treating children living with HIV should work in close collaboration with infectious disease doctors familiar with these medications. Infants with perinatal HIV-1 infection should be treated with ART as soon as the diagnosis is established. Children with microalbuminuria or proteinuria should be followed more closely to prevent the progression of kidney diseases. In addition, ACE inhibitors (ACEi) appear to provide therapeutic benefits in adults and are recommended to treat proteinuria in children with HIV kidney diseases [73] (Table 2). For more detailed information regarding the specific treatment doses and ART regimens for children and adolescents with HIVAN, we recommend following the guidelines for the management of CKD in patients with HIV [73], as well as the guidelines for the use of antiretroviral agents in children and adolescents living with HIV [74].

**Table 2:** General guidelines for the treatment of children and adolescents with HIVAN

*Treatment goal:* achieve virological suppression causing the least possible systemic and renal toxicity	
*Treatment team:* always include nephrologists and HIV-infectious diseases experts	
*Preferred ART regimen*: two nucleoside reverse transcriptase inhibitors (NRTIs) plus one drug from one of the following classes: integrase inhibitors (INSTI), non-nucleoside reverse transcriptase inhibitors (NNRTI), or a boosted protease inhibitor (PI). The regimen should be simple to assure adherence	
*Choice of regimen:* should take into consideration the patient’s age, body weight, sexual maturity, results of drug-resistance testing, dosing frequency, food or fluid requirements, pill size, availability of palatable pediatric formulations, drug interactions, and preference of the patients and caregivers	
*Doses and regimen selection:* recommend following the guidelines of the “Panel on ART and medical management of children and adolescents living with HIV, which are updated frequently when the FDA approves new drugs and regimens: http://aidsinfo.nih.gov/con- tentfiles/lvguidelines/pediatricguidelines	
*Co-infections:* Children and adolescents with tuberculosis or hepatitis should receive specific ART regimens, as discussed in the guidelines above	
*Proteinuria and hypertension:* use doses of ACE inhibitors (ACEI) or angiotensin receptor blockers (ARB) recommended for children with other kidney diseases or hypertension	
*Renal toxicity:* monitor frequently by doing urinalysis, and assessing the SCr levels at least once a year. Be aware of the ARV drugs that cause renal toxicity, and adjust their doses in children with CKD	
*Infants and young children:* are at risk of developing diarrhea, dehydration, or salt wasting disorders. The treatment with ACEI and ARB should be monitored more frequently	
*Goal in adolescents*: keep HIV RNA levels below 200 copies/ml, to prevent the transmission of HIV to sexual partners. Use drugs that do not cause fetal damage, and monitor patients treated with ACEI/ARB	

#### Kidney transplantation and HIVAN

Kidney transplantation of children with HIV-CKD has become an accepted treatment modality in recent years; however, significant outcome data in children are still lacking**.** Very promising results reported in pilot safety studies were performed in highly selected groups of HIV+ kidney transplant recipients [75]. These highly selected HIV+ kidney recipients showed excellent adherence to ART therapy, lack of severe immunosuppression (CD4 > 200 cells/μl), undetectable viremia (< 50 HIV-1 RNA copies/ml) for 3 months prior to the kidney transplant, absence of AIDS-defining illness, successful immune reconstitution after ART, no history of opportunistic infections or neoplasms, and no active viral infections for at least 6 months previous to the kidney transplant. More studies done in the USA and Europe confirmed that kidney transplantation is a safe and effective treatment for kidney failure in HIV+ patients [75, 76], with equivalent patient and allograft survival to uninfected patients [75]. Unique challenges for HIV+ patients, however, include the high rates of acute rejection, delayed graft function, and significant drug–drug interactions [75]. In a prospective study including 150 kidney transplant recipients with undetectable plasma HIV-1 RNA, there was no histological evidence of recurrent de novo FSGS on light microscopy, nor was there any evidence of nephrotic range proteinuria [76]. Moreover, recurrent HIV nephropathy was not a factor in early graft loss (within 3 years of transplant in the USA trial). Unfortunately, very few pediatric nephrology transplant programs worldwide have transplanted a significant number of HIV+ children, and it is advisable to follow the adult guidelines. Finally, the limited availability of kidney transplants for children raises ethical concerns. To address this issue, surgeons in South Africa are pursuing kidney transplants using HIV+ kidney donors with good results [77]. Based on these data, in October 2016, surgeons at Johns Hopkins performed the first liver and kidney transplant in the USA between an HIV+ donor and HIV+ positive recipient and an ongoing clinical trial is assessing the outcome of more patients.

#### Perspectives

During the last 36 years, great progress has been made in our understanding of the pathogenesis and treatment of children and adolescents with HIVAN. The quality of life and survival of children and adolescents living with HIV has improved in a remarkable manner and the prevalence of HIV-associated kidney diseases has decreased significantly. Nonetheless, many children living with HIV are reaching adulthood with proteinuria, mild low GFR levels, and at high risk of developing kidney failure later in life. Furthermore, we continue to see children with HIVAN and other HIV-associated kidney diseases in the Washington DC area. A number of challenges remain for the appropriate treatment of children living with HIV during the post-cART era, including the long-term persistence of viral reservoirs and inflammation resulting in end organ and kidney damage. From the renal pathological point of view, more work needs to be done to understand the mechanisms through which HIV-1 infects and injures kidney epithelial cells, define the cell types and mechanism modulating the regeneration and recovery of kidney epithelial cells, determine whether kidney epithelial and/or infiltrating mononuclear cells could become a reservoir for the virus, define the role of the APOL-1 risk variants in the pathogenesis of childhood HIVAN, and explore other potential risk factors associated with African ancestry. From the clinical and epidemiological point of view, more work needs to be done to develop a vaccine, prevent the vertical transmission of HIV-1, provide appropriate ART regiments to all children, maintain their viral suppression throughout childhood and adolescence, develop programs to identify children and adolescents undergoing the early stages of proteinuria, and define the safety and renal toxicity of new ART, as well as the outcomes of kidney transplants in children. These are all big healthcare challenges that will require the coordinated effort of people working in multiple disciplines. Hopefully, better prophylactic treatments and vaccines will be developed in the near future to achieve the ultimate goal of eradicating this disease.
